# Case report: Pneumonia with clinical symptoms precedes imaging evidence after immune checkpoint inhibitors combined with radiotherapy in lung squamous cell cancer

**DOI:** 10.3389/fimmu.2022.998516

**Published:** 2022-09-15

**Authors:** Yao Wang, Yimeng Wang, Jinming Yu, Xiangjiao Meng

**Affiliations:** ^1^ Department of Radiation Oncology and Shandong Provincial Key Laboratory of Radiation Oncology, Shandong Cancer Hospital and Institute, Shandong First Medical University and Shandong Academy of Medical Sciences, Jinan, China; ^2^ Research Unit of Radiation Oncology, Chinese Academy of Medical Sciences, Jinan, China

**Keywords:** immune checkpoint inhibitors (ICI), immune-related adverse event(irAE), pneumonitis, programmed cell death 1 inhibitor, cancer immunotherapy

## Abstract

Immune-checkpoint inhibitors (ICI) targeting programmed cell death 1 (PD-1) and its ligand 1 (PD-L1) have quickly changed the treatment landscape in advanced non-small cell lung cancer. However, any patient treated with an immune checkpoint inhibitor is at risk for immune-related adverse events (irAEs). Checkpoint inhibitor pneumonitis (CIP) is a rare but potentially severe pulmonary toxicity of immunotherapy. Since the imaging features and symptoms are not specific, the diagnosis of CIP is challenging. In addition, CIP may mimic other lung diseases. Due to these characteristics, proper patient management may be delayed. So, a comprehensive understanding of imaging features is essential for a prompt detection and correct management of these drug-induced lung diseases. We presented a patient with lung squamous cell cancer who has clinical symptoms preceding imaging evidence of pneumonitis after immunotherapy and radiotherapy. We also discussed the safety of immunotherapy, the complexity and management of immune pneumonitis.

## Introduction

Immune-checkpoint inhibitors (ICI) have transformed the treatment of multiple cancer, which significantly improved survival (1–3). One of the remarkable characteristics of ICIs is their relatively mild toxicity profile. Although uncommon, immune-related adverse events (irAEs) may occur in patients receiving immunotherapy, especially in those receiving combined ICI. The mechanisms leading to Checkpoint inhibitor pneumonitis (CIP) are still being clarified. Some potential mechanisms include increasing T-cell activity in response to cross-antigens, increasing levels of autoantibodies and inflammatory cytokines and enhancing complement mediated inflammation (4). Most of irAEs are low grade and can involve almost any organ system. They have uncertain features, but in most cases can be improved by drug immunosuppression and discontinuation of treatment. CIP is uncommon but potentially fatal toxicity of immunotherapy.

The clinical manifestations of CIP are variable, it might show asymptomatic disease, or it may present with respiratory symptoms such as cough, shortness of breath and respiratory failure, in some cases can lead to death. The incidence of CIP in combined therapy (6.5%–10%) was higher than that in monotherapy (3%–4%) (4–6). However, due to the lack of typical imaging findings, the radiological diagnosis of CIP is challenging. In most cases, the main radiologic patterns of CIP include several abnormalities with cryptogenic organizing pneumonia (COP), nonspecific interstitial pneumonitis (NSIP), hypersensitive pneumonitis (HP), ground-glass opacities, and acute interstitial pneumonitis for more severe cases. In some cases, CIP can display a special imaging feature that cannot be classified in any of the above-mentioned specific modes (7). Moreover, CIP is a dynamic process that evolves over time. Most studies reflect the incidence of CIP, but do not clarify the course of it. Here, we report a patient with CIP whose imaging features lag behind clinical symptoms after immunotherapy and radiotherapy.

## Case presentations

**Figure 1:**
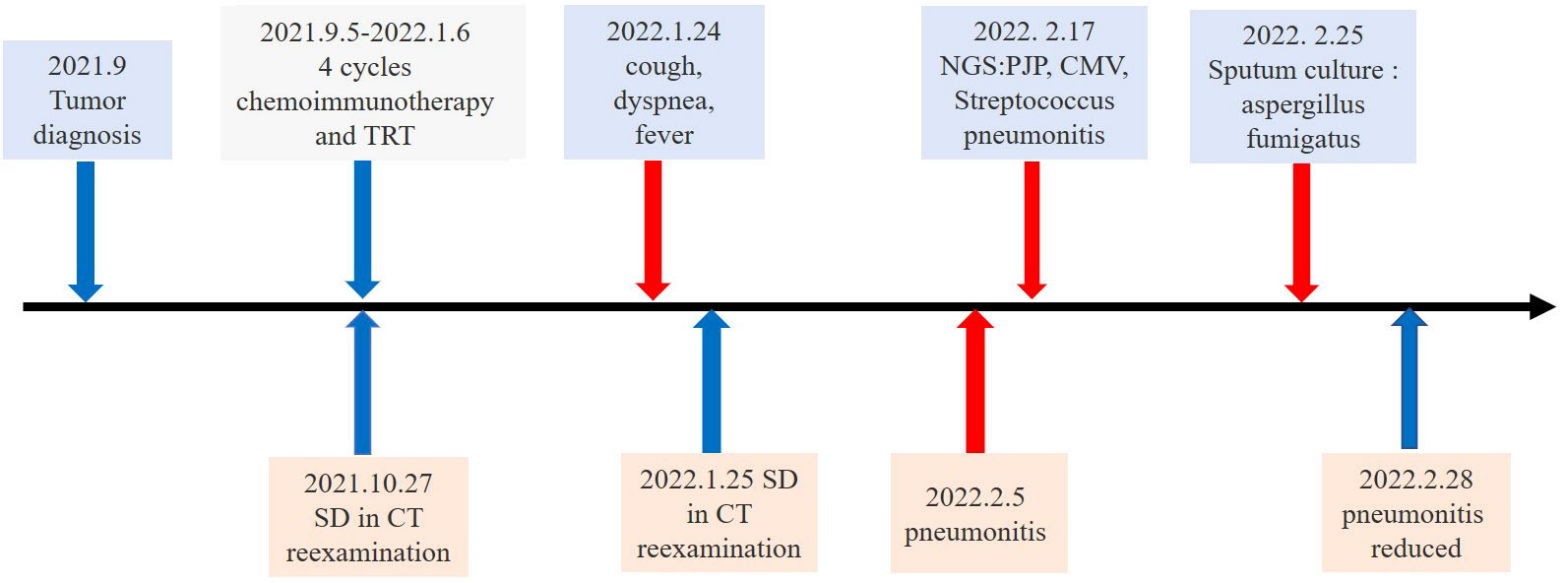
Timeline of the major treatment process and CT evaluation of the patient since diagnosis. SD means “stable disease”.

**Figure 2:**
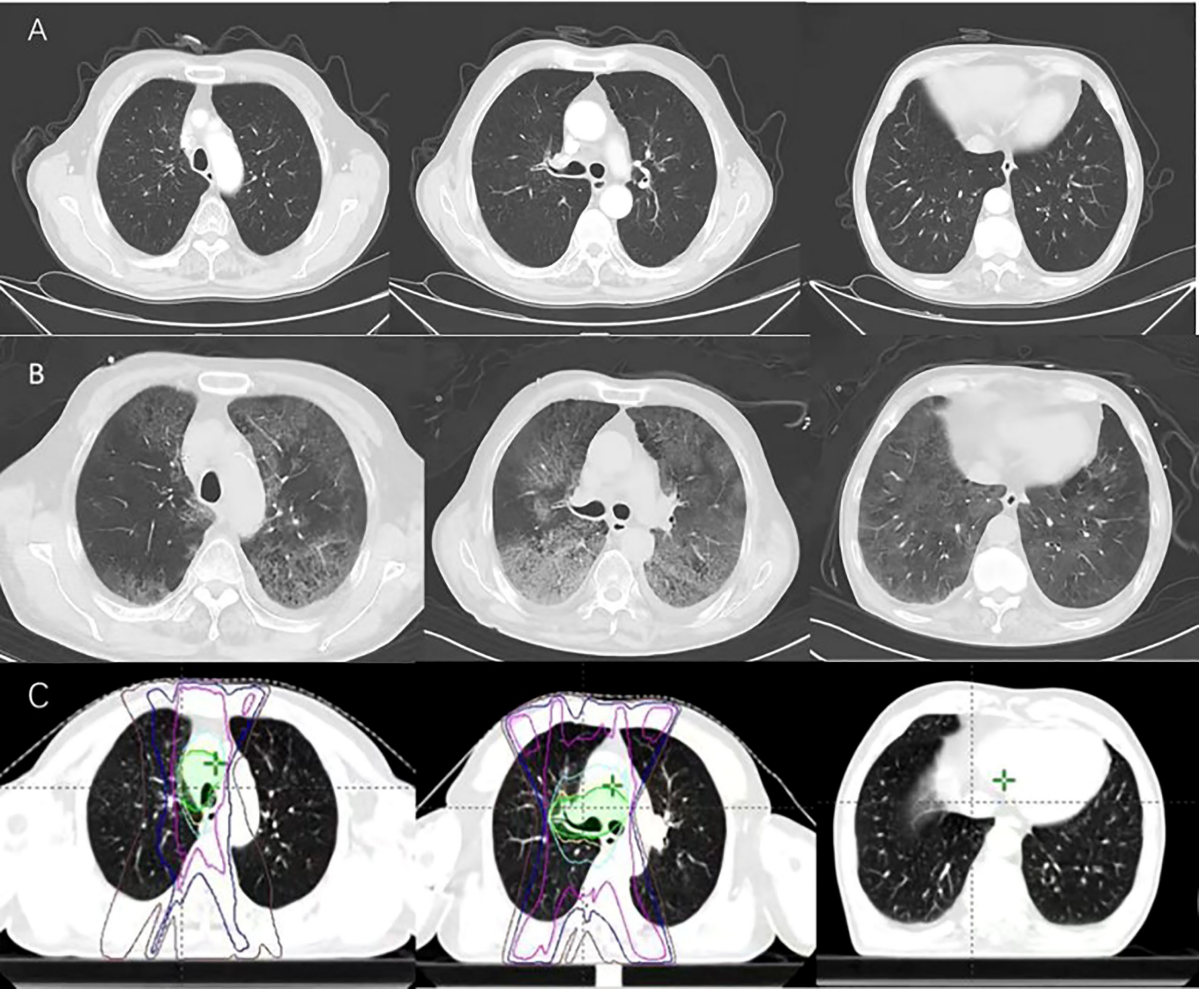
**(A)** 20 days after treatment ends, dyspnea, cough and fever occurred. **(B)** 27 days after treatment ends. **(C)** Radiation field.

A 66-year-old man with stage IIIIC lung squamous cell carcinoma (SCC) (cT4N3M0) presented with cough and chest pain for four months (**Figure 1**). He had a history of smoking for more than 40 years (40 cigarettes a day, quit > 3 months). His body weight was 71 kg, with no significant change from the previous weight. No sensitive gene mutations were detected and the percentage of tumor cells with membranous PD-L1 staining (tumor proportion score) was 30%. Four cycles of tislelizumab plus nab-paclitaxel and carboplatin chemotherapy were performed. The fourth cycle of treatment were synchronized with thoracic radiotherapy (TRT) (60 Gy/30 fractions), the mean lung dose (MLD) was 8.07 Gy, 22.5% of the lung received 20 Gy (V20), and 36% of the lung received 5 Gy (V5), which ended in Jan 2022 (**Figure 2C**). The patient’s symptoms of cough and chest pain improved.

After 20 weeks of the initial tislelizumab administration (20 days after radiotherapy ended), dyspnea and cough developed. The patient presented to our hospital, the laboratory tests including hemogram and inflammatory markers were normal except for the white blood cell of 14.79×10^9^/L. Computerized tomography (CT) of the chest showed the tumor was reduced in size and no pneumonitis was observed (**Figure 2A**). We administered an inhaled acetylcysteine solution for the symptomatic treatment of dyspnea. However, 1 week later, the patient had a fever with a worsening cough and expectation. Due to the Spring Festival, he did not seek medical attention in time. Then his temperature continued to rise. Only 3 days later, in the emergency department, CT was notable for extensive exudation of both lungs, diagnosis of interstitial pneumonia (**Figure 2B**). The patient’s condition continued to deteriorate and he was transferred to the intensive care unit (ICU). His temperature was 42°C, the pulse was 123 beats per minute, mask oxygen was given to the patient. Then his partial pressure of oxygen (PaO2) was only 60 mmHg and the fraction of inspiration O2 (FiO2) was 57%. Serum (1–3)-ß-d-glucan level was 327.12pg/mL, and the galactomannan was 0.25μg/L. According to the multidisciplinary team (MDT), the patient had a history of previous immunotherapy. CT showed large blurred shadow with unclear margins, and his serum (1–3)-ß-d-glucan level are high. It was thought to be pulmonary complications related to immune checkpoint inhibitor therapy and possible coinfection. Then methylprednisolone (40 mg per 12 hours) was administered intravenously. And antibiotics such as Compound Sulfamethoxazole Tablets (Trimethoprim 0.16g and Sulfamethoxazole 0.8g per 6 hours), Sulperazon (3g per 8 hours) and voriconazole (0.2g per 12 hours) were empirically administered. After a week, the patient’s condition did not improve, and voriconazole was replaced by caspofungin (70mg once→50mg/day). Bronchoscopy with bronchoalveolar lavage (BAL) was performed. On the 12th hospital day, next-generation sequencing (NGS) analysis of BALF specimens identified Pneumocystis jirovecii(PJP), streptococcus pneumonia and cytomegalovirus (CMV). Ganciclovir (375mg/day for seven days → 0.25g per 12 hours) was added to his treatment and increased the dose of Compound Sulfamethoxazole Tablets (Trimethoprim 0.24g and Sulfamethoxazole 1.2g per 6 hours). At the same time, the patient with sustained grade III/IV thrombocytopenia, immunoglobulin (25 g/day for five days) was injected intravenously. On the 20th hospital day, the fungal and bacterial sputum culture obtained 7 days earlier grew Aspergillus fumigatus. The patient improved after 23 days in ICU and CT showed reduced inflammation.

## Discussion

### Occurrence of CIP

We described a case of CIP after radiotherapy combined with immunotherapy whose symptoms predate imaging findings, complicated by Pneumocystis, Aspergillus and other infections during the treatment of CIP. Previous studies have shown that during PD-1 inhibitor monotherapy, the incidence of all-grade pneumonia in NSCLC was 4.1%, and the incidence of grade 3 and above pneumonia was 1.8% (6). ICIs may overstimulate the immune system, change the homeostasis of the host, and lead to excessive inflammatory response. During radiotherapy, ionizing radiation induces free radicals and DNA damage, promoting oxidative stress, vascular damage, and inflammation on normal tissues (8). A pool analysis showed, about 11% of patients who received stereotactic body radiotherapy (SBRT) developed grade ≧̸2 radioactive pneumonia within a few months (9). The combination of radiation therapy(RT) and ICI works not only by directly tumor-cell killing, but also by stimulating the immune system response through radiation, so as to enhance the ICI effect (10). Many preclinical studies have shown that there is synergistic activity between ICIS and RT. The combination of thoracic radiotherapy and ICI may have synergistic pulmonary toxicity. The occurrence of pneumonitis is an important point worth exploring.

Some studies have demonstrated the safety of radiotherapy combined with immune checkpoint inhibition. In the KEYNOTE-001 trial, the secondary analysis found that prior thoracic radiotherapy was associated with an increased risk of treatment-related pulmonary toxicities (13% vs. 1%, P = 0.046), while there was no significant difference in high-grade pulmonary toxicity (11). The phase 3 PACIFIC trial showed the incidence of any grade pneumonitis/radiation pneumonitis was 33.9% in the immunotherapy group and 24.8% in the placebo group (P value not reported), and the incidence of grade 3 or higher pneumonitis/radiation pneumonitis occurred in 3.4% and 2.6%, respectively (P value not reported) (12). They mainly demonstrated the pulmonary toxicities of sequential combination of ICI with a previous history of radiotherapy. A recent retrospective study in patients who underwent TRT after ICI evaluated the incidence and severity of treatment-related pneumonitis. It showed that the incidence and severity of treatment-related pneumonitis was significantly higher in lung cancer patients who received TRT after ICI (13). As is known, acute “radiation pneumonitis” usually occurs within 4 to 12 weeks after thoracic irradiation. Recently, some studies revealed that pneumonitis in radio-immunotherapy patients usually occurs in previously irradiated areas, regardless of how much time has elapsed after RT, even after a few years. In some patients receiving ICIS, the time interval between radiation therapy and pneumonitis raises the hypothesis of “radiation recall pneumonitis”. A recent pooled analysis found that the patients received radiotherapy within 90 days (RT ≤ 90) after ICI had slightly numerically higher rates of pneumonitis than those more than 90 days (RT >90) before the start of ICI therapy. These differences were due to low grade (grade 1-2) AEs (14). In addition, a phase 1 trial of Pembrolizumab combination with chemoradiotherapy for locally advanced NSCLC found pneumonitis of ≥grade 2 occurred in 33% patients, and risk of pneumonitis was higher in patients who started pembrolizumab on day 1 of chemotherapy (15). In summary, the combination of ICI and radiotherapy have a slightly higher rate of pneumonitis than monotherapy, but the difference may be owing to low grade (grade 1-2) AEs pneumonitis that is mostly tolerated. In addition, timing of combination therapy is critical. Longer intervals seem to portend better security. If the interval exceeds 90 days, there is no significant increase in the probability of pneumonitis.

### Complex diversity of CIP

The most common symptoms of pneumonitis are dyspnea and cough, fever and chest pain were less common, some patients were asymptomatic at the onset of pneumonitis (16). Several cases of CIP have been reported, indicating that the clinical course of pneumonitis also varies among patients, with some requiring ICU admission, intubation and even death, while others are successfully treated with oral corticosteroid. In addition, a few patients experience recurrent pneumonitis after restarting their immunotherapy (5, 7, 17). Retrospective case studies have inconsistently identified risk factors for underlying lung disease, such as ILD, history of radiation therapy, history of COPD and V20. However, the factors involved are highly variable for individual patients (18).

As far as we know, we present the first case of patient’s pneumonitis symptoms prior to radiologic manifestation. In this case, when the patient returned to the hospital with cough, dyspnea, and fever, a CT scan on him found no obvious signs of pneumonitis. However, 1 week later, his symptoms gradually worsened and CT also showed severe pneumonitis. The patient was treated with methylprednisolone (80mg per 12 hours) and empirically administered with antibiotics. His condition had not significantly improved but found coinfection. Regarding infectious complications, it has emerged that the blockade of the PD-1/PD-L1 axis does not appear to inherently increase the risk of infection because it promotes T-cell effector functions. Nevertheless, they can induce irAEs. The treatment of these irAEs require additional immunosuppressive with corticosteroids which can lead to opportunistic infection (19). However, unlike the majority of reported cases, the new reports showed that some infections can be triggered by ICIs without immunosuppressive treatment. It seems that hyperinflammatory dysregulated immunity associated with ICIs drives pathogenesis. These infections are characterized by a dysregulated inflammatory immune response (20).

The patient's NGS analysis also found CMV, there is evidence that CMV may be a potential trigger for severe irAE. A retrospective cohort study comparing CMV infection in patients with different degrees of CIP showed that the CMV positive rate was much higher in patients with severe CIP than in patients with no or mild ICI pneumonitis (91.7 vs. 20%). There are reports that immunotherapy exacerbates progressive fungal infections, such as aspergillosis, in the absence of immunosuppression. Clinically, this infection can mimic the progression of cancer. Therefore, immune checkpoint inhibition may exaggerate the immune response to fungal colonization, which may promote fungal growth (21).

With rare exceptions, CIP may also be accompanied by other irAE, such as this patient developed thrombocytopenia with normal hemoglobin and normal white cell counts. Bone marrow biopsy showed no obvious morphological abnormalities, no hemophagocytic cells, and no malignant invasion in the patient. Laboratory tests such as antinuclear antibodies were negative, but antiphospholipid and antiplatelet antibodies were abnormal. Thrombocytopenia caused by chemotherapy, infection, or other drugs, was excluded, and the final diagnosis was immune-induced thrombocytopenia. While steroid therapy, he received five platelet transfusions and intravenous immunoglobulin (25 g/day for five days), but the platelets did not recover. In the meantime, the lowest platelet level was 7 × 10^9^/L. Fortunately, his platelets returned to normal after discharge. This also reminds us that in the face of patients after immunotherapy, the adverse reactions may be complex, and we should comprehensively evaluate and designate a comprehensive and individualized treatment plan.

CIP is essentially interstitial pneumonia and opportunistic infections are prone to occur in CIP patients. However, early diagnosis is difficult due to the acute course of infection and the heterogeneity of clinical manifestations. Bronchoscopy and bronchoalveolar lavage (BAL) are key to confirming infection, and NGS should be performed if necessary. We should pay attention to distinguishing between bacterial infection, viral infection, fungal infection and heart failure. Occasionally, patients may present with symptoms without imaging findings, which deserves our vigilance.

### Diagnosis and treatment of CIP

The diagnosis of CIP is exclusionary, which is usually a combination of clinical assessment, imaging findings, and laboratory analysis. BAL analysis is also crucial as it can differentiate inflammation and tumor. The BAL cytology shows a predominantly lymphocytic or a mixed pattern (7). Inflammatory markers such as C-reactive protein were also elevated. In some patients who can’t have a BAL, imaging findings are crucial to the diagnosis of disease. In imaging, the mainly differential diagnoses of CIP are infection and tumor progression, which can share similar clinical symptoms. The relationship between the radiation field and pneumonia deserves our attention.

To date, the treatment of irAEs has mostly been empirical. Several published guidelines recommended similar treatments for different grades of pneumonitis. Grade 1 pneumonitis is treated by discontinuation of ICI therapy without steroids. Grade 2 pneumonitis can be managed by withholding the ICI therapy and initiating steroids therapy. Grades 3 and 4 pneumonitis should permanent discontinuation of ICI therapy and intravenous steroids along with empirical antibiotic therapy. Therefore, the most common initial treatment for CIP is steroids. But a small proportion of patients are refractory or become resistant to steroids, which is rare but is associated with higher morbidity and mortality (5, 22). For these patients, treatment guidelines suggest second-line immunosuppressive therapy including infliximab, mycophenolate, intravenous immunoglobulin and cyclophosphamide (23–25). Since the clinical course and response pattern to steroids are often individualized. The timing of initiation of additional immunomodulatory agents varies among patients. At present, it is considered that a more effective treatment strategy is to give additional immunomodulatory agents as soon as possible once the patient has the initial signal of refractory response (26). Other promising strategies, such as targeting the microbiome, are emerging and have yet to be included in these guidelines. Multiple case reports of opportunistic infections after ICI treatment by a variety of different pathogens proved the necessity of having a low threshold of investigation for opportunistic infection (20).

As for patients who develop CIP, it has been demonstrated that the use of high-dose glucocorticoids increases the risk of pneumocystis. Thus, PJP prophylaxis has to be considered (27). Increased vigilance and timely identification of pneumonia are prerequisites for early initiation of treatment and prevention of further morbidity and mortality in these patients (28). Because CIP has no clear onset time, patients may have no imaging findings, and even a few patients have symptoms prior to imaging findings. It is recommended that continues to strengthen the monitoring of patient symptoms and signs and laboratory results during immunotherapy (29).

The case has several limitations. The main limitation is due to the serious condition of the patient, methylprednisolone was given before there have a BAL analysis or other microbiology result, which may interfere the BAL cytology. Antibiotics are also used empirically, which prevents us from accurately assessing the initial infection status. In addition, we lack the serial blood gas analysis of the patients, which prevent us to understand the treatment response through PaO2/FiO2. Given these limitations, more CIP patients deserve our attention.

## Conclusion

In patients with CIP, a new feature that the clinical symptoms predate imaging findings deserves our attention. CIP is gradually increasing following the use of immune checkpoint inhibitors. When thoracic radiotherapy is combined with ICI, timing of combine is critical, longer intervals seem to portend better security. Clinicians should strengthen the surveillance and management of pneumonitis. Opportunistic infections are prone to occur in CIP patients and steroid therapy should be used earlier when pulmonary toxicity occurs. It has been demonstrated that the use of high-dose glucocorticoids increases the risk of PJP. Thus, PJP prophylaxis has to be considered. There are critical needs to clarify the mechanisms of CIP and to formulate individualized treatment strategy to improve the safety of tumor immunotherapy. Looking into the future, radiologists must strengthen collaboration with multidisciplinary teams to provide optimal treatment and management for patients with ICI-associated pneumonia, which is so complicated in diagnosis and treatment.

## Data availability statement

The original contributions presented in the study are included in the article/supplementary material. Further inquiries can be directed to the corresponding authors.

## Ethics statement

Written informed consent was obtained from the individual for the publication of any potentially identifiable images or data included in this article.

## Author contributions

XM proposed, edited, and approved the final manuscript. MY collected clinical information. YW analyzed the data and drafted the manuscript. All authors contributed to the article and approved the submitted version.

## Funding

This work was supported by the Academic Promotion Program of Shandong First Medical University (2019ZL002), Research Unit of Radiation Oncology, Chinese Academy of Medical Sciences (2019RU071), the foundation of National Natural Science Foundation of China (81627901, 81972863 and 82030082), the foundation of Natural, Science Foundation of Shandong (ZR201911040452). Natural Science Foundation of Shandong (ZR2019MH010), CSCO-Haosen Foundation (Y-HS202102-0089), and CSCO-Xinda Foundation (Y-XD202001-0008).

## Conflict of interest

The authors declare that the research was conducted in the absence of any commercial or financial relationships that could be construed as a potential conflict of interest.

## Publisher’s note

All claims expressed in this article are solely those of the authors and do not necessarily represent those of their affiliated organizations, or those of the publisher, the editors and the reviewers. Any product that may be evaluated in this article, or claim that may be made by its manufacturer, is not guaranteed or endorsed by the publisher.
